# Current update on the visual outcome of optic pathway glioma associated with neurofibromatosis type-1

**DOI:** 10.3389/fsurg.2022.908573

**Published:** 2022-09-02

**Authors:** Janice Lasky Zeid

**Affiliations:** ^1^ Division of Ophthalmology, Ann & Robert H. Lurie Children's Hospital of Chicago, Chicago, IL, United States; ^2^ Department of Ophthalmology, Feinberg School of Medicine, Northwestern University, Chicago, IL, United States

**Keywords:** optic pathway glioma, neurofibromatosis type 1, optic pathway tumor, diagnosis, treatment

## Abstract

**Purpose:**

Clinical and diagnostic evaluation in the follow-up of optic glioma patients with neurofibromatosis type 1 (NF-1) can be difficult. Determining whether and when to provide treatment is a significant challenge in best managing these patients. Update on current information and future directions in management is included in this review.

**Current Practice:**

NF-associated optic pathway gliomas (OPGs) present a significant management challenge with high stakes for visual outcomes. Monitoring vision and diagnostic tests are challenging in patients of a younger age. Regardless of whether few or many optic gliomas are encountered during clinical practice.

**Summary:**

This review of optic gliomas-NF1-associated gliomas includes the current approach and knowledge of OPG-NF1 and future directions in OPG-NF1 management. This includes the ongoing Multicenter Natural History Study and other clinical trials and outcomes in NF-1 patients with OPG.

## Current knowledge of patients with OPG-NF1

Neurofibromatosis type 1 (NF1) is one of the most common genetic diseases worldwide, with an incidence of approximately 1:3,500 births (1). It is autosomal dominant, with half of the cases representing sporadic mutations (2). It is caused by a mutation in the tumor suppressor gene of NF1. Optic pathway glioma (OPG) is the most common orbital and intracranial manifestation of NF1, typically presenting prior to 6 years of age, although symptomatic tumors have been reported in older children (3). OPG-NF1 is generally benign, with approximately half to two-thirds of OPG-NF1 patients having minimal tumor progression. One-third to half may have significant morbidity, mainly vision loss and endocrine abnormalities (4–6). A number of reports have included both NF1-associated OPGs and sporadic OPGs. In contrast, sporadic OPGs (not associated with NF1) progress more frequently and have a worse prognosis (7–9). Any portion of the anterior and posterior visual pathway, optic nerves, chiasm, optic tract, and hypothalamus may be affected by OPG-NF1 (10–12).

The largest multicenter retrospective study to date, based in the United States, looking at vision after chemotherapy for NF1-associated OPG included 115 patients (13). Our center participated in this study. Of these 115 patients, 88 patients and 168 eyes could be evaluated for visual acuity (VA) outcome. It is important to note that at the completion of chemotherapy, VA improved in 22% of eyes (32% of subjects), remained stable in 57% of eyes (40% of subjects), and declined in 21% of eyes (28% of subjects). Approximately one-third of children regained some VA with treatment. In addition, tumor location does convey outcome; the more posterior tumors are more likely to progress. Posterior location predisposes to worse vision outcomes with involvement of the optic tracts/radiations with comparably worse visual outcomes. In addition, there is a poor correlation between radiographic and VA outcomes. Essentially, it was shown that there was no correlation between vision progression and tumor progression on MRI. It follows that we need to consider both MRI and vision to determine the best management. It is also interesting that in every study of OPG-NF1, there are more girls than boys, and girls have more progression.

Now, 10 years have passed since the conduct of the study mentioned above, and a recent study that looked at a large group of children in Portugal with OPG-NF1 produced similar results (14) (Table 1). The primary aim of this study was to analyze visual outcomes by comparing children who underwent chemotherapy with those who did not receive treatment. The association of visual outcomes with retinal nerve fiber layer (RNFL) thickness and tumor location was also studied. Forty-one percent received chemotherapy. In this study, children with OPG-NF1 in the non-treated group, when best-corrected VA at the last follow-up was measured, 73% of eyes were stable, and 27% improved. In the chemotherapy group, 62% were stable, 13% improved, and 25% worsened. Therefore, either vision improved or was stable in all eyes in the non-treated group and in 75% of eyes in the chemotherapy group. Sixty-one percent of patients were female. It may be a self-fulfilling prophecy that those requiring chemotherapy had fewer eyes that remained stable or improved. It may be expected that patients requiring chemotherapy would not do as well, although overall, most patients did well. The conclusions were that children treated with chemotherapy had worse visual outcomes, although treatment stabilized or improved vision in 75% of patients. In treated patients, retrochiasmatic tumors appeared to do worse.

**Table 1:** Comparison of visual outcomes of optic pathway glioma in neurofibromatosis treated with chemotherapy.

VA at completion of chemotherapy	Fisher et al.^a^ (13) % of eyes	José et al. (14) % of eyes
Improved VA	22	13
Stable VA	57	62
Worse VA	21	25

VA, visual acuity.

^a^ Did not study non-treated eyes.

Our group recently published a long-term follow-up study in children with OPG-NF1, which is the longest follow-up that we are aware of (15). We included 45 children with 10 years of follow-up or more. Previous reports of follow-up periods have been relatively short (6–9, 16, 17). In this 10-year or greater follow-up, in multivariate analysis, the VA and optic nerve appearance at presentation predicted final outcomes. All patients, except for one who was asymptomatic at presentation and who had normal VA and normal optic nerve appearance, went on to have good vision in both eyes. Forty percent needed treatment, and the average time from OPG-NF1 diagnosis to treatment was 10 months. At the end of follow-up, almost 90% kept good vision (20/30 or better) when both eyes were open. Moderate-to-severe impairment is defined as 20/40 or worse in both eyes. Almost two-third kept good vision in their worse eye. Sixty-four percent had good VA per eye. This indicated that children with OPG-NF1 who had a normal initial examination had excellent long-term visual and anatomical outcomes (Table 2). The VA and appearance of the optic nerve head at presentation predicted long-term outcomes.

**Table 2:** Visual outcomes of optic pathway glioma in neurofibromatosis after long-term follow-up of at least 10 years (15).

VA after long-term follow-up^a^	Initial exam % of eyes	End of follow-up % of eyes	Initial exam per subject^b^	End of follow-up per subject^b^
Normal	65	64	87	89
Abnormal	35	36	13	11

Normal VA was considered mild to no impairment (20/20–20/30). Abnormal VA was considered moderate-to-severe impairment (20/40 or worse).

^a^ Includes both treated and untreated eyes.

^b^ Binocular vision (both eyes open).

## Vision screening for OPGS in NF1 patients

The findings of the above recent studies support decision-making to minimize screening neuroimaging of asymptomatic patients as long-term prognosis is good. Although some authors may support early diagnosis of OPG-NF1 by screening MRI prior to the development of symptoms to hopefully lead to improved vision outcomes (18), others suggest that early diagnosis of asymptomatic OPG may not justify the adverse effects of repeated sedation for MR imaging (3, 16, 19). The OPG-NF1 task force in 1997 determined no conclusive evidence that early detection of asymptomatic gliomas would reduce the vision loss rate (20). In our practice, we do not perform the screening of MRIs. Imaging with MR is performed if indicated by a reduced vision for age, optic nerve abnormalities such as edema or pallor, or other visual concerns.

The preferred screening eye examination protocol for asymptomatic children with NF1 is variable to the institution. Children with NF1 aged 6 years and under are at the greatest risk for symptomatic OPG-NF1 (3, 16, 20–23), although *de novo* symptomatic OPG-NF1 or previously known OPG-NF1 with progression may manifest in older children and during adulthood (3, 6, 24). The American Academy of Pediatrics recommends ophthalmic examination annually from the age of diagnosis to under 8 years of age, followed by a complete eye examination every 2 years (25). In our practice, we perform yearly examinations until the age of 10 and with an increased interval after that. Examinations should be repeated or performed more frequently if there are any visual concerns.

VA assessment for screening examinations and optimal follow-up of known OPG can be challenging in children. In very young children, qualitative measures such as fix and follow or central steady and maintenance will be assessed. Quantitative assessment of vision in patients 6 months or older is optimal. Teller acuity card (TAC) testing is possible for children of 6 months and older. Matching LEA pictures or HOTV optotypes is possible in children 3 years or older. This is a crucial part of the examination, and having a skilled tester is important. Deterioration in vision can be defined as a two-line difference in VA. If the reduced vision cannot be attributed to an anatomic problem of the eye, refractive error, amblyopia, or lack of cooperation, then imaging or repeat imaging with MRI is warranted.

Visual field testing is an additional visual measurement that should be attempted in cooperative children. Basic confrontation field testing should be performed at office examinations using finger counting or toys as feasible. In patients with known optic gliomas, kinetic visual field testing is helpful. Goldman visual field kinetic testing may be performed, as it is easy to perform for young children and is more suitable for them, but the disadvantage of wide test–retest variability still persists in this testing method (3). It is important to be cautious in interpreting results and repeating testing if changes are seen in this subjective testing method.

Ocular coherence tomography (OCT) is a newer modality used to evaluate children with OPG. Peripapillary RNFL thickness, as measured by OCT in children 6 years or older with OPGs, was found to be decreased in most children who had abnormal VA and or a visual field (VF) defect (26). Avery et al. looked at the longitudinal changes in circumpapillary RNFL measured by spectral-domain (SD)-OCT in children with OPG, both with and without NF (27). The findings indicated a decline greater than or equal to 10% circumpapillary RNFL thickness in one or more quadrants, or the global average was predictive of vision loss. The absence of decline was predictive of stable vision. Global average and inferior quadrant were the best predictive values. It was suggested that circumpapillary RNFL may be used as a surrogate marker of vision and can be helpful in taking management decisions, particularly when young children are unable to cooperate with quantitative VA or visual field testing. Overall, how OCT should be utilized has yet to be fully studied. In our practice serving a large NF clinic, we may repeat OCT in an interval of 3–6 months early on in the course of OPG. If a decrease in OCT is seen and yet there is no change in vision, we consider repeating OCT in 1–2 months to confirm a decrease in RNFL thickness which may indicate progressive damage from OPG. We find it another helpful parameter to use in monitoring OPG-NF1.

## Optic pathways’ involvement of OPG-NF1 as a prognostic factor

OPG-NF-1 may affect any portion of the visual pathway. OPG-NF1 was bilateral in about 34.8% of patients in one study (18). It is important to recognize that tumor location does convey outcome, with the more posterior tumors more likely to progress. The largest multicenter trial in the United States to date found that tumor involvement in the optic tracts/radiations is a consistent prognostic indicator for poor visual outcome (13). A recent study in Portugal noted that children in the retrochiasmatic glioma group (Dodge stage 3) appeared to have worse visual outcomes (14). Young age and postchiasmal involvement for OPG-NF1 are associated with a worse visual prognosis despite treatment (4, 10, 13, 28). This is helpful in considering the risk for progression in managing children with OPG-NF1.

## Timing and decision-making in the treatment of OPT-NF-1

Optimal timing and treatment decisions are controversial, and multiple factors are involved. Children with OPG-NF-1 have, overall, good visual outcomes. Children treated with chemotherapy for OPG-NF1 experienced worse visual outcomes, as you may expect, but it stopped the decline of vision or improved vision in approximately 70% of patients (13, 14). In our practice, we consider treatment for progressive vision loss. Serial eye examinations by a skilled practitioner measuring quantitative vision are important to determine if two lines or more of reduction are present and/or reproduced. Treatment consideration is indicated to stabilize and prevent further vision loss. Improvement in vision may be seen in one-third or less of patients. In addition, there is a poor correlation between radiographic and VA outcomes. Fisher et al. showed no correlation between vision progression and tumor progression on MRI (13). One needs to consider both MRI and vision to determine the best management. Progression of OPG on MRI would indicate a need for repeat imaging and close follow-up by eye examination. Treatment for progression on MRI may be considered, especially with large changes and/or patients having difficulty in obtaining a quantitative vision (TAC testing, LEA symbols, HOTV, and Snellen). It is helpful to consider that the posterior location of OPG may lead to worse visual outcomes. The lack of correlation between visual and MRI outcomes argues against using imaging response as the main outcome of treatment success.

## Future research on the management of NF-associated OPG

Important to enhancing OPG-NF1 knowledge or to future research is a currently ongoing prospective observational multicenter study, “Developing Evidence-Based Criteria for Initiating Treatment for Neurofibromatosis Type 1 Associated Optic Pathway Glioma” (ongoing and unpublished). To gain good insight it is the power of numbers; we are currently joining this multicenter prospective trial in the United States that aims to recruit 250 children with NF1 and newly diagnosed OPG. There is an observational cohort and a treatment cohort. Michael Fisher and Robert Avery are the study chairs for this NF1-OPG Natural History Study. The primary goal is to determine the prognostic factors for visual outcome for newly diagnosed OPG-NF1. Secondary goals include determining prognostic factors for imaging progression, for visual outcome, and for imaging outcome in the treatment group, and assessing the correlation between visual and imaging outcomes. Ancillary studies looking at optical coherence tomography (OCT) and Goldman visual field in patients in this patient population are also ongoing. This important multicenter trial will help answer questions surrounding the natural history of OPG-NF1.

Currently, chemotherapy is the first-line treatment modality for OPG in most centers (3, 10, 29, 30). The combination of vincristine and carboplatin is effective in controlling progressive and recurrent gliomas (31, 32). Carboplatin is now considered a standard treatment for OPG-NF1 (11). Carboplatin hypersensitivity reaction can occur in up to 40% of patients (33, 34). Vinblastine has been used in place of carboplatin/vincristine in hypersensitivity reactions (33). In some centers, it may be used as initial treatment.

Important to the future of chemotherapy treatment is a multicenter trial looking at Selumetinib. This mitogen-activated extracellular signal regulated kinase (MEK) inhibitor has been used previously in plexiform neurofibromas to compare with standard treatment and determine visual outcomes in patients with OPG-NF1. “A phase 3 Randomized Study of Selumetinib Versus Carboplatin/Vincristine in Newly Diagnosed or Previously Untreated NF1 Associated Low-Grade Glioma (LGG)” (NCT03871257, unpublished). Selumetinib is a mitogen-activated protein kinase 1 and 2 (MEK1/2) inhibitor, a drug that is a kinase inhibitor (blocks a key enzyme) that OPG tumor cells need for growth. The use of Selumetinib will compare with standard treatment carboplatin/vincristine and determine visual outcomes.

## Summary of management of NF1-associated OPG

NF-associated OPG may present a significant management challenge with high stakes for visual outcomes. Monitoring vision and diagnostic tests are challenging in patients of a younger age. Overall, most patients with OPG-NF1 have good visual outcomes. Observation is possible in most cases with careful monitoring of vision. If there is evidence of progressive vision loss, it may indicate a need for chemotherapy treatment even if MR imaging does not show tumor progression as the two do not necessarily correlate. MR imaging is important in monitoring tumor changes. The newer modality of OCT is helpful in following patients and can be an early indicator of RNFL changes that may affect vision. Current multicenter trials will shed light on questions and controversies about OPG-NF1 management and treatment.

## Author contributions

JLZ wrote and edited the manuscript. All authors contributed to the article and approved the submitted version.

## References

[1] Friedman JM Gutmann DH MacCollin M Riccardi VM. Neurofibromatosis: phenotype, natural history, and pathogenesis. Baltimore: Johns Hopkins University Press (1999).

[2] Lázaro C Ravella A Gaona A Volpini V Estivill X. Neurofibromatosis type 1 due to germ-line mosaicism in a clinically normal father. N Engl J Med. (1994) 331(21):1403–7. 10.1056/NEJM199411243312102 7969279

[3] Listernick R Ferner RE Liu GT Gutmann DH. Optic pathway gliomas in neurofibromatosis-1: controversies and recommendations. Ann Neurol. (2007) 61(3):189–98. 10.1002/ana.21107 17387725 PMC5908242

[4] Balcer LJ Liu GT Heller G Bilaniuk L Volpe NJ Galetta SL Visual loss in children with neurofibromatosis type 1 and optic pathway gliomas: relation to tumor location by magnetic resonance imaging. Am J Ophthalmol. (2001) 131(4):442–5. 10.1016/S0002-9394(00)00852-7 11292406

[5] Listernick R Darling C Greenwald M Strauss L Charrow J. Optic pathway tumors in children: the effect of neurofibromatosis type 1 on clinical manifestations and natural history. J Pediatr. (1995) 127(5):718–22. 10.1016/S0022-3476(95)70159-1 7472822

[6] Thiagalingam S Flaherty M Billson F North K. Neurofibromatosis type 1 and optic pathway gliomas: follow-up of 54 patients. Ophthalmology. (2004) 111(3):568–77. 10.1016/j.ophtha.2003.06.008 15019338

[7] Dodgshun AJ Elder JE Hansford JR Sullivan MJ. Long-term visual outcome after chemotherapy for optic pathway glioma in children: site and age are strongly predictive. Cancer. (2015) 121(23):4190–6. 10.1002/cncr.29649 26280460

[8] Kalin-Hajdu E Décarie JC Marzouki M Carret AS Ospina LH. Visual acuity of children treated with chemotherapy for optic pathway gliomas. Pediatr Blood Cancer. (2014) 61(2):223–7. 10.1002/pbc.24726 23956233

[9] Varan A Batu A Cila A Soylemezoğlu F Balcı S Akalan N Optic glioma in children: a retrospective analysis of 101 cases. Am J Clin Oncol. (2013) 36(3):287–92. 10.1097/COC.0b013e3182467efa 22547006

[10] Liu GT. Optic gliomas of the anterior visual pathway. Curr Opin Ophthalmol. (2006) 17(5):427–31. 10.1097/01.icu.0000243016.90004.12 16932058

[11] Liu GT Brodsky MC Phillips PC Belasco J Janss A Golden JC Optic radiation involvement in optic pathway gliomas in neurofibromatosis. Am J Ophthalmol. (2004) 137(3):407–14. 10.1016/j.ajo.2003.09.055 15013861

[12] Savar A Cestari DM. Neurofibromatosis type I: genetics and clinical manifestations. Semin Ophthalmol. (2008) 23(1):45–51. 10.1080/08820530701745223 18214791

[13] Fisher MJ Loguidice M Gutmann DH Listernick R Ferner RE Ullrich NJ Visual outcomes in children with neurofibromatosis type 1-associated optic pathway glioma following chemotherapy: a multicenter retrospective analysis. Neuro-Oncology. (2012) 14(6):790–7. 10.1093/neuonc/nos076 22474213 PMC3367846

[14] José P Couceiro R Passos J Jorge Teixeira F. Visual outcomes of optic pathway glioma treated with chemotherapy in neurofibromatosis type 1. J Pediatr Ophthalmol Strabismus. (2022) 59(2):128–35. 10.3928/01913913-20210720-02 34592874

[15] Kinori M Armarnik S Listernick R Charrow J Zeid JL. Neurofibromatosis type 1-associated optic pathway glioma in children: a follow-up of 10 years or more. Am J Ophthalmol. (2021) 221:91–6. 10.1016/j.ajo.2020.03.053 32283094

[16] Segal L Darvish-Zargar M Dilenge ME Ortenberg J Polomeno RC. Optic pathway gliomas in patients with neurofibromatosis type 1: follow-up of 44 patients. J AAPOS. (2010) 14(2):155–8. 10.1016/j.jaapos.2009.11.020 20451859

[17] Wan MJ Ullrich NJ Manley PE Kieran MW Goumnerova LC Heidary G. Long-term visual outcomes of optic pathway gliomas in pediatric patients without neurofibromatosis type 1. J Neuro-Oncol. (2016) 129(1):173–8. 10.1007/s11060-016-2163-4 27311725

[18] Prada CE Hufnagel RB Hummel TR Lovell AM Hopkin RJ Saal HM The use of magnetic resonance imaging screening for optic pathway gliomas in children with neurofibromatosis type 1. J Pediatr. (2015) 167(4):851–6.e1. 10.1016/j.jpeds.2015.07.001 26233602 PMC9100836

[19] Ing C DiMaggio C Whitehouse A Hegarty MK Brady J von Ungern-Sternberg BS Long-term differences in language and cognitive function after childhood exposure to anesthesia. Pediatrics. (2012) 130(3):e476–85. 10.1542/peds.2011-3822 22908104

[20] Listernick R Louis DN Packer RJ Gutmann DH. Optic pathway gliomas in children with neurofibromatosis 1: consensus statement from the NF1 optic pathway glioma task force. Ann Neurol. (1997) 41(2):143–9. 10.1002/ana.410410204 9029062

[21] Listernick R Charrow J Greenwald M Mets M. Natural history of optic pathway tumors in children with neurofibromatosis type 1: a longitudinal study. J Pediatr. (1994) 125(1):63–6. 10.1016/S0022-3476(94)70122-9 8021787

[22] Listernick R Charrow J Greenwald MJ Esterly NB. Optic gliomas in children with neurofibromatosis type 1. J Pediatr. (1989) 114(5):788–92. 10.1016/S0022-3476(89)80137-4 2497236

[23] Zeid JL Charrow J Sandu M Goldman S Listernick R. Orbital optic nerve gliomas in children with neurofibromatosis type 1. J AAPOS. (2006) 10(6):534–9. 10.1016/j.jaapos.2006.03.014 17189147

[24] Listernick R Ferner RE Piersall L Sharif S Gutmann DH Charrow J. Late-onset optic pathway tumors in children with neurofibromatosis 1. Neurology. (2004) 63(10):1944–6. 10.1212/01.WNL.0000144341.16830.01 15557519

[25] Hersh JH. Health supervision for children with neurofibromatosis. Pediatrics. (2008) 121(3):633–42. 10.1542/peds.2007-3364 18310216

[26] Avery RA Liu GT Fisher MJ Quinn GE Belasco JB Phillips PC Retinal nerve fiber layer thickness in children with optic pathway gliomas. Am J Ophthalmol. (2011) 151(3):542–9.e2. 10.1016/j.ajo.2010.08.046 21232732 PMC3053008

[27] Avery RA Cnaan A Schuman JS Trimboli-Heidler C Chen CL Packer RJ Longitudinal change of circumpapillary retinal nerve fiber layer thickness in children with optic pathway gliomas. Am J Ophthalmol. (2015) 160(5):944–52.e1. 10.1016/j.ajo.2015.07.036 26231306 PMC4661093

[28] Opocher E Kremer LC Da Dalt L van de Wetering MD Viscardi E Caron HN Prognostic factors for progression of childhood optic pathway glioma: a systematic review. Eur J Cancer. (2006) 42(12):1807–16. 10.1016/j.ejca.2006.02.022 16809032

[29] Gandhi NG. Treatment of neuro-ophthalmic and orbitofacial manifestations of neurofibromatosis type 1. Curr Opin Ophthalmol. (2013) 24(5):506–11. 10.1097/ICU.0b013e32836348a4 23846187

[30] Mahoney DH Jr Cohen ME Friedman HS Kepner JL Gemer L Langston JW Carboplatin is effective therapy for young children with progressive optic pathway tumors: a pediatric oncology group phase II study. Neuro-oncology. (2000) 2(4):213–20. 10.1093/neuonc/2.4.213 11265230 PMC1920597

[31] Packer RJ Ater J Allen J Phillips P Geyer R Nicholson HS Carboplatin and vincristine chemotherapy for children with newly diagnosed progressive low-grade gliomas. J Neurosurg. (1997) 86(5):747–54. 10.3171/jns.1997.86.5.0747 9126887

[32] Packer RJ Lange B Ater J Nicholson HS Allen J Walker R Carboplatin and vincristine for recurrent and newly diagnosed low-grade gliomas of childhood. Am J Clin Oncol. (1993) 11(5):850–6. 10.1200/JCO.1993.11.5.850 8487049

[33] Lafay-Cousin L Holm S Qaddoumi I Nicolin G Bartels U Tabori U Weekly vinblastine in pediatric low-grade glioma patients with carboplatin allergic reaction. Cancer. (2005) 103(12):2636–42. 10.1002/cncr.21091 15861409

[34] Yu DY Dahl GV Shames RS Fisher PG. Weekly dosing of carboplatin increases risk of allergy in children. J Pediatr Hematol Oncol. (2001) 23(6):349–52. 10.1097/00043426-200108000-00005 11563768

